# Binafuxi granules in the treatment of common cold with heat syndrome based on traditional Uighur medicine: study protocol for a multicenter randomized controlled trial

**DOI:** 10.1186/s13063-019-3290-y

**Published:** 2019-03-29

**Authors:** Jie Min, Bin She, Xin Zhang, Bing Mao, Yan Chen

**Affiliations:** Pneumology Group, Department of Integrated Traditional and Western Medicine, West China Hospital of Sichuan University, 37 Guoxue Lane, Chengdu, 610041 Sichuan Province People’s Republic of China

**Keywords:** Common cold, Traditional Uighur medicine, Heat syndrome, Binafuxi granules, Randomized controlled trial

## Abstract

**Background:**

The common cold is a highly prevalent illness with significant impact on society and health care. Common cold with heat syndrome (CCHS) is one of the most common types based on syndrome differentiation by traditional Uighur medicine (TUM), which is widely used in Central Asia. The study is designed to explore the efficacy, safety and optimal therapeutic dosage of Binafuxi granules in treating CCHS.

**Methods:**

This is a multicenter, randomized, double-blind, placebo-controlled, phase II clinical trial. Participants (*n* = 240) will be enrolled from five centers across China and randomly assigned to the high-dose group, low-dose group or placebo control group in a 1:1:1 ratio. All eligible patients will receive test drugs twice daily for 3 days. The primary outcome is the time to fever relief. Secondary outcomes include the time to fever clearance, duration of primary symptoms and each symptom and change in TUM symptom score.

**Discussion:**

This is the first placebo-controlled randomized clinical trial of a Uighur medicine in treating common cold. It will provide robust evidence on the efficacy and safety of Binafuxi granules in the treatment of CCHS.

**Trial registration:**

Chinese Clinical Trial Registry, ChiCTR-IIR-17013379. Registered on 14 November 2017.

**Electronic supplementary material:**

The online version of this article (10.1186/s13063-019-3290-y) contains supplementary material, which is available to authorized users.

## Background

The common cold is a highly prevalent, upper respiratory tract, infectious disease, mainly caused by more than 200 viruses [1]. Varying among different viruses, the symptoms of the common cold often include sneezing, runny nose, nasal congestion, sore throat, coughing, fever and general malaise [2, 3]. The duration of these symptoms is generally 3–7 days, but is occasionally up to several weeks [1]. Despite being generally mild, the common cold still has a significant impact on society and health care [4]. According to previous data, the common cold is the third most common diagnosis in physician visits [5]. It even can be a trigger for severe or even fatal illness in individuals with chronic respiratory diseases or other underlying conditions [6].

So far, the treatment of common cold is aimed at symptom relief. Complementary and alternative medicine (CAM), primarily herbal and nutritive remedies, has become very popular recently [7]. Many clinical studies of CAM, such as on the use of echinacea, vitamin D, vitamin C, probiotics, ginseng and Chinese herbal medicine, have demonstrated efficacy and safety in treating the common cold [8–14]. Traditional Uighur medicine (TUM) is the common traditional medicine used in Xinjiang Uygur Autonomous Region, China. With a history of more than 2500 years, it is based on the theory of ancient Uighur medicine, combining the theories of traditional Chinese medicine (TCM) and ancient Greek, Egyptian, Arabian and Indian medicine. The humorism, one of the most important TUM theories, is based on the four materials (fire, air, water and earth) and the four *mizaj* (temperaments) (hot, cold, moist and dry) [15], and the humorae theory indicates that the *hilit* (body fluid) is composed of *kan* (blood), *belghem* (phlegm), *sapra* (yellow bile), and *savda* (black bile) [16]. Based on humorism, the common cold, which is also called *zukamu* in TUM, can be divided into heat syndrome and cold syndrome. The common cold with heat syndrome (CCHS) is generally related to the imbalance status and abnormal change in *kan* and *sapra* in the four body fluids [17]. CCHS is primarily characterized by fever (body temperature > 38 °C), sore throat, nasal congestion, nasal discharge, cough and headache.

The Binafuxi is an Uighur herbal medicine prescription and derived from ancient books on Uighur medicine, including *Mahzinul Murakkibat* (Uighur Combination Medicine) and *Karabadin Azam*. It has been used for treating the CCHS for more than 300 years. The prescription is mainly composed of tianshanjincai (*Viola tianshanica Maxim*), heguotenggen (roots of *Operculina turpethum*), gancaojingao (extractum Glycyrrhizae), meiguihua (*Flos rosae rugosae*), sikamoniyazhi (*Resina scammoniae*) and alihong (*Sclerotium fomitis officinalis*). Binafuxi granules are based on the Binafuxi prescription and have been approved by the State Food and Drug Administration of China (SFDA) for a clinical trial (approval number 2014 L01342). Unpublished pharmacologic experiments have demonstrated that Binafuxi granules relieve fever, alleviate cough and sweating and reduce inflammation. The data showed that Binafuxi granules can significantly reduce endotoxin-induced fever in rabbits administered clinical equivalent doses (0.52 g/kg) of Binafuxi granules. Additionally, there was no chronic toxicity found in rats administered different doses of Binafuxi granules (30, 15 and 7.5 times the normal dose) for 1 month except for some cases of diarrhea that occurred in the group receiving 30 times the normal dose.

Since ancient times, many patients have benefited from TUM treatment of the common cold. However, to our knowledge there have been no high-quality clinical studies of the use of TUM in treating the common cold. In conformity with the Drug Administration Law of the People’s Republic of China and good clinical practice (GCP) issued by the SFDA, we designed this randomized clinical trial to evaluate the efficacy and safety of Binafuxi granules in patients with CCHS and to detect the optimal therapeutic dosage.

## Methods/design

**Fig. 1:**
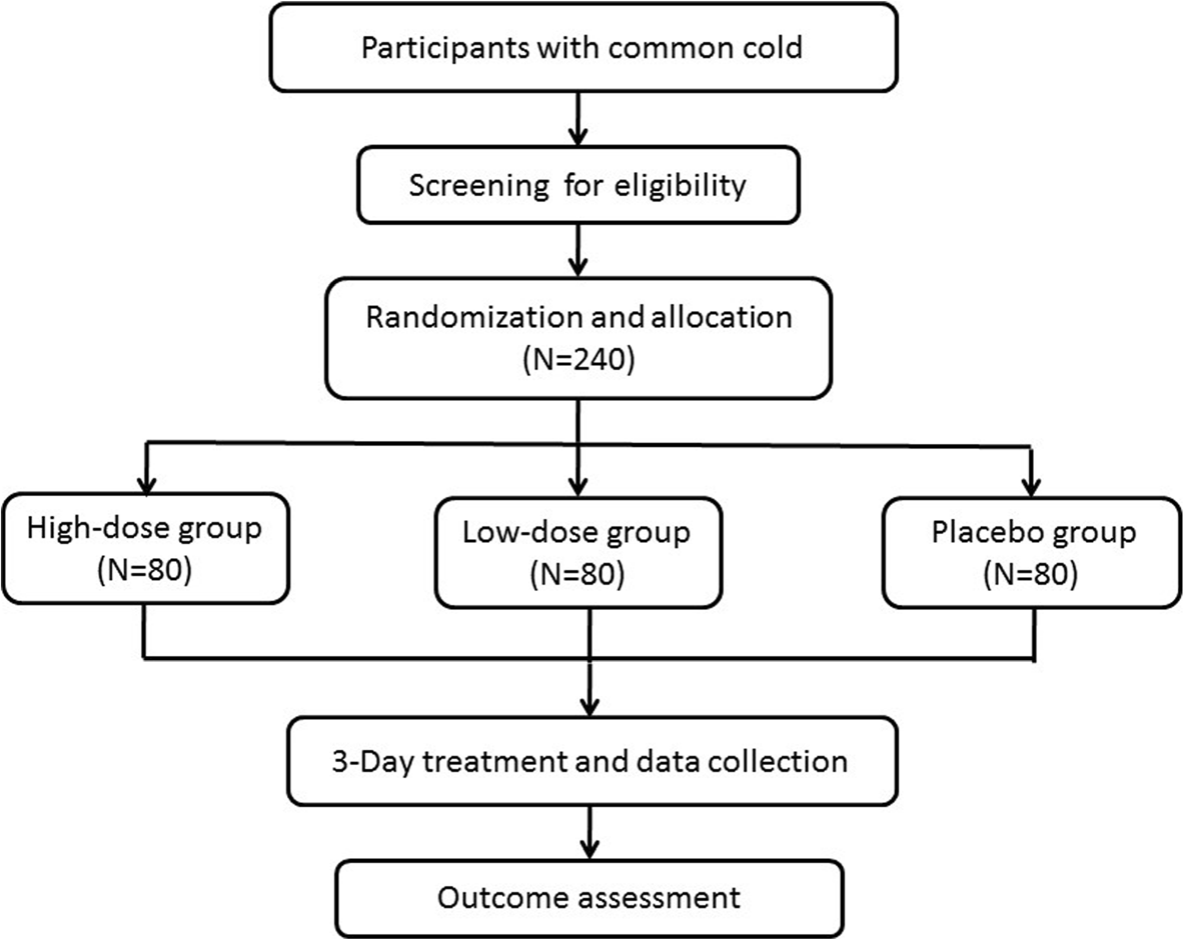
Flowchart. We will recruit 240 patients who will randomly be allocated to one of three groups (high-dose group, *n* = 80; low-dose group, *n* = 80; control group, *n* = 80). After 3 days, we will analyze and assess the data. TUM, traditional Uighur medicine

This trial is a multicenter, double-blind, placebo-controlled and randomized phase II clinical study, which has been authorized by the SFDA (approval number: 2014 L01342) and registered with the Chinese Clinical Trial Registry (ChiCTR-IIR-17013379). The study is financially supported by Xinjiang Yinduolan Uighur Medicine Co. Ltd., Xinjiang, China, which had or will have no role in the study design, study conduct, data management or decision to submit the study results. This trial will be conducted in five trial sites in China. A total of 240 patients will be enrolled and randomly allocated to the high-dose group, low-dose group or placebo control group, in a 1:1:1 ratio. Binafuxi granules and placebo will be provided for 3 days to subjects in the three study groups, respectively. The study flow chart is shown in Fig. 1.

The trial protocol has been approved by the Ethics Committee of Clinical Trials and Biomedicine of West China Hospital of Sichuan University (IRB-2017-5). All eligible patients have to provide their signed informed consent prior to enrollment. The privacy of all subjects will be properly protected. The personal information on eligible patients will be concealed by identification codes and all records will be stored in a locked location.

### Recruitment

By means of advertisements and recommendations, we recruit participants at the following five research sites across China: (1) West China Hospital of Sichuan University, (2) Shanghai Traditional Chinese Medicine Hospital, (3) Xinjiang Traditional Chinese Medicine Hospital, (4) Ruikang Hospital Affiliated to Guangxi University of traditional Chinese medicine and (5) Lishui People’s Hospital. Each site will recruit patients in equal numbers.

Eligible participants in these five sites will be enrolled by the well-trained investigators. The investigators will talk with eligible patients about the study and obtain informed consent from them. The Principal Investigator is responsible for subject recruitment in each site.

### Inclusion criteria

The inclusion criteria are as follows: (1) meet the diagnosis of common cold according to Western medicine; (2) meet the diagnosis of CCHS according to traditional Uighur medicine; (3) fever and body temperature between 38 and 39 °C; (4) symptom presenting within 24 h; (5) age between 18 and 65 years and (6) voluntarily participate and provide signed informed consent.

### Exclusion criteria

The exclusion criteria are as follows: (1) influenza, acute or chronic rhinitis, acute sinusitis, suppurative tonsillitis, pneumonia and tuberculosis; (2) white blood cell count > 11.0 × 10^9^/L and/or neutrophil percentage > 75%; (3) taking any medication to treat common cold; (4) liver function levels (alanine aminotransferase (ALT) and aspartate aminotransferase (AST)) 1.5 times higher than the upper limit of normal or abnormal serum creatinine; (5) serious primary cardiovascular, pulmonary, kidney, liver, neurological or hematological disease; (6) pregnant, lactating or planning to become pregnant; (7) allergic or possibility of being allergic to the ingredients in the study drug; (8) participating in or have participated in other clinical drug trials within the last 3 months and (9) identified by the investigator as inappropriate to participate in this study.

### Diagnostic criteria

Diagnosis of the common cold in Western medicine is established according to the diagnostic criteria for the common cold of acute upper respiratory tract infection of *Internal Medicine, 8*^*th*^*Version* (2013) [18]. The typical clinical symptoms of common cold include sneezing, nasal congestion, nasal discharge, fever and sore throat.

The TUM diagnosis of CCHS is based on the Clinical research guidelines for treatment of common cold with new Uyghur medicine (2017) [17]. The TUM diagnostic criteria for CCHS include primary symptoms, secondary symptoms, signs for the tongue and signs for the pulse. The primary symptoms include fever, nasal congestion, nasal discharge and sore throat. The secondary symptoms include cough, headache, thirst, sore limbs and sweating. The signs for the tongue are red tongue and thick yellow tongue coating. The signs for the pulse are hard and rapid pulse. To meet the diagnosis of CCHS, patients should have at least two of the primary symptoms in which fever is essential, and at least two of the secondary symptoms, as well as the TCM signs for the tongue and pulse.

### Interventions

**Fig. 2:**
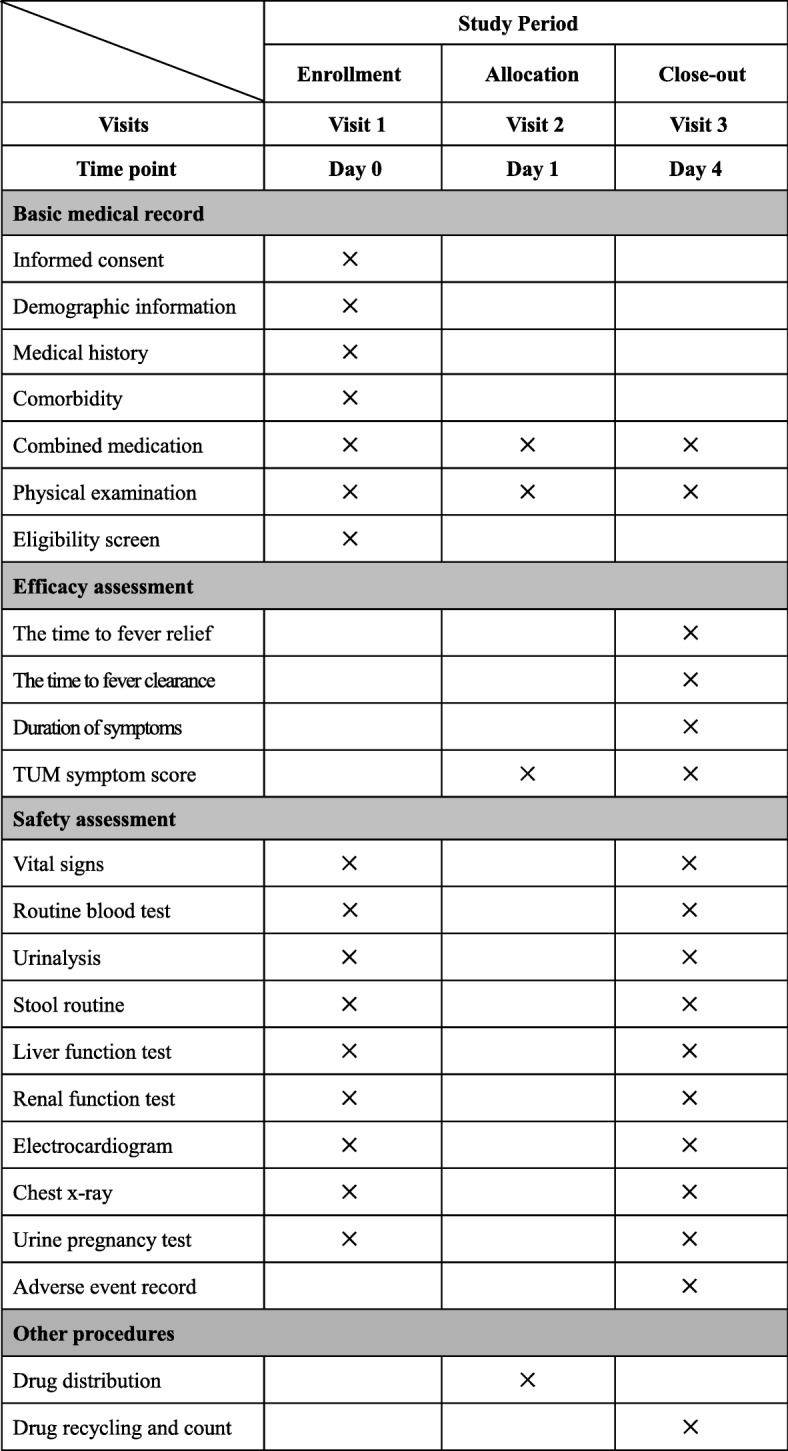
Study schedule and Standard protocol items: recommendation for interventional trials (SPIRIT) figure

Binafuxi granules (batch Y170521) and placebo granules (batch Y170520) are manufactured by Xinjiang Yinduolan Uighur Medicine Co. Ltd., Xinjiang, China. The minimum effective dose of Binafuxi granules is 2.75 g. The Chinese herbal medicine placebo consists of 20-fold dilution of 2.75 g of Binafuxi granules and maltodextrin with the addition of artificial pigment and flavoring agents [19]. Patients in the placebo group will be administered placebo granules, which are almost identical to the Binafuxi granules in appearance, smell and taste. All test drugs are concealed in unified sealed packages and each package contains a 2.75-g dose. In each site, an independent drug administrator is responsible for dispensing, reclaiming, storing and recording of all test drugs. Patients in the high-dose group, low-dose group or placebo group will receive two packages of Binafuxi granules (5.5 g) twice daily, one package of Binafuxi granules (2.75 g) plus one placebo package (2.75 g) twice daily, or two placebo (5.5 g) twice daily, respectively. All test drugs will be dissolved in warm water and taken orally two times daily for 3 days. Throughout the trial, participants will be visited at baseline and on the fourth day post baseline (see Fig. 2). The study protocol complies with Standard Protocol Items: Recommendations for Interventional Trials (SPIRIT) (see Additional file 1). We will not regularly follow up all patients except in the case of an adverse event.

### Concomitant treatments and forbidden drugs

During the study, if the body temperature of a patient rises above 39.0 °C and/or the body temperature do not drop significantly at 48 h post administration, paracetamol can be used to bring down fever following the physician’s advice. Patients can continue their treatments for underlying conditions, such as diabetes mellitus or hypertension. Rescue treatment in the case of drug-related adverse events is also permitted in this study. Furthermore, considering the related impact on the study results, a patient with a drug-related adverse event may be withdrawn from this trial. All concomitant medications or treatments in the study must be recorded carefully in the case report form (CRF). Any other medication or therapy for common cold is not allowed during the study.

### Randomization and blinding

According to a stratified block randomization method, 240 patients are stratified by study center and randomly assigned to the high-dose group, low-dose group or placebo control group in a 1:1:1 ratio. The random sequence and the randomization list of each center will be generated by the independent specialist, using the PRCO PLAN function of the analysis system of SAS software (SAS, Cary, NC, USA). The independent drug administrator will assign numbered packs of study drugs to eligible patients in order by randomization list. The randomization sequence will be concealed in a lightproof sealed envelope, which will be kept by the leader and the Sponsor.

Participants, investigators and statisticians will be blind to the treatment allocation throughout the study. Test drugs and placebo will be identical in appearance, color and taste. All drugs are concealed in uniform packages with number labels. An emergency letter including the random sequence and assignment has been prepared in each center. In any emergency medical situation, such as serious adverse event or deteriorative condition, the unblinding process will be started after contacting the Sponsor and the primary investigator. The investigator should record the details of urgent unblinding and make sure the corresponding patient is excluded.

### Outcome measures

#### Primary outcomes

The primary outcome of the study is the time to fever relief, which is defined as the time (number of hours) from the first dose of the test drug until the axillary temperature drops by at least 0.5 °C. All patients are required to record their axillary temperature in the subject diary every 1 h within the first 6 h. Then, if the temperature is ≥ 37.3 °C, it should be recorded every 2–4 h, and if the temperature is < 37.3 °C, it should be recorded at 8 a.m. and 4 p.m. every day. If the temperature is < 37.2 °C and does not rise for 24 h, it does not need to be recorded. The investigator will check and collect the subject diary in which the data will be acquired and evaluated on the fourth day.

#### Secondary outcomes

Secondary outcomes include the time to fever clearance, duration of primary symptoms and each symptom and change in TUM symptom score.

##### Time to fever clearance

The time to fever clearance is defined as the time (number of hours) from the first dosing to the axillary temperature dropping to < 37.2 °C and no longer rising for 24 h.

##### Duration of all symptoms and each symptom

The duration of all symptoms is defined as the number of hours from enrollment to the time that all symptoms completely disappear. Each patient is required to record any change in symptoms in the subject diary. The investigator will assess the duration of all symptoms and each symptom.

##### Change in TUM symptom score

Currently, there is no grading evaluation standard for Uighur medicine. In this trial, the TUM symptom graded score system follows the *Guidelines for clinical research on new drug of chinese medicine* [20] (see Table 1). The primary symptoms are given a graded score (none = 0, mild = 3, moderate = 6, severe = 9) and the secondary symptoms are given a graded score (none = 0, mild = 1, moderate = 2, severe = 3). TUM signs will also be assessed, but not scored. The total score of TUM symptoms (including primary and secondary symptoms) will be summed at baseline (symptom score before treatment) and the fourth day (symptom score after treatment). The sum of all symptom scores is the cumulative TUM symptom score. The change in cumulative TUM symptom score is assessed by the percentage of symptom score reduction (PSSR), which is calculated according to the following formula:

$$ \mathrm{PSSR}=\left(\frac{symptom\ score\ before\ treatment- symp\mathrm{t} om\  score\ after\ treatment}{symptom\ score\ before\ treatment}\right)\times 100\% $$

**Table 1:** Symptom scoring system and TUM sign

TUM Symptoms	Score			
Primary	0	3	6	9
Fever	None	37.3 °C–37.9 °C	38 °C–38.5 °C	>38.5 °C
Nasal congestion	None	Mild	Moderate	Severe
Nasal discharge	None	Occasionally	Sometimes	Frequently
Sore throat	None	Dry throat with slight pain	Significant with obvious pain when swallowing	Sore and swollen throat with severe pain
Secondary	0	1	2	3
Cough	None	Occasionally in daytime	Occasionally day and night	Frequently day and night
Headache	None	Slight and occasionally	Slight and lasting	Severe and affecting working
Thirsty	None	Dry mouth	Moderate	Severe and drinking plenty of water
Sweating	None	Mild	Moderate	Severe
Sore limbs	None	Slight	Moderate	Severe
Cumulative symptom score	Primary symptom scores + secondary symptom scores			
TUM sign	Unscored			
Tongue body	□Red	□Others:		
Tongue coating	□Thick and yellow	□Others:		
Pulse condition	□Hard and rapid	□Others:		

*TUM* traditional Uighur medicine

Based on the therapeutic effect evaluation system, the improvement of TUM symptoms will be categorized into clinical recovery (PSSR ≥ 70%) and invalid (PSSR < 70%).

### Safety assessment

Safety assessment, including physical examination and laboratory tests, will be performed at baseline and the fourth day. Physical examination includes vital signs (temperature, respiration, heart rate and blood pressure) and conventional examination (chest, abdomen and nervous system, etc.). Laboratory tests include routine blood test, urine analysis, stool test, measurement of blood electrolytes, liver function test (ALT, AST, alkaline phosphatase (ALP), total bilirubin (TBil) and gamma-glutamyl transpeptidase (GGT)) and renal function test (blood urea nitrogen (BUN), serum creatinine, microalbuminuria, urinary nag enzyme and serum cystatin C). In addition, before treatment, all patients are required to undergo a chest x-ray and electrocardiogram, and female patients of reproductive age are required to take a urinary pregnancy test.

All adverse events (AEs) should be truly recorded in detail on the adverse event form (AEF) and followed up to completion. The patient experiencing an AE will be treated appropriately until recovery from it. A serious AE (SAE) is defined as an adverse event resulting in death, a life-threatening event, an illness requiring hospitalization or severe disability, and it should be recorded and reported to the Principal Investigator, SFDA, ethics committee and the Sponsor within 24 h.

### Quality control

As the monitoring center, Beijing QiHuang Clinical Drug Research Center is in charge of monitoring implementation of the trial protocol, the CRF data and the safety of the participants, as well as contacting and coordinating the various units. The center will assign the Clinical Research Associate (CRA) to regularly review the CRFs and monitor the row data every week. The CRA is blind to trial assignment. Monitoring results should be presented to the Principal Investigator in each site. The Principal Investigator will be responsible for the implementation of the trial, which should be following the standard operation procedure (SOP) for quality assurance and quality control. Any protocol modification should be documented with appropriate justification and approved by the Sponsor, primary investigator in each unit and statisticians. Then the final version should be presented to the Ethics Committee, and the SFDA if required.

### Sample size calculation

Using “One-Way Analysis of Variance F-Tests using Effect Size” in PASS (version 15.0.5), we estimate the sample size with a significance level of 0.05. To our knowledge, no previous study has reported the effective size related to the time to fever relief in our target population (patients with common cold). Thus, we used η2 as an alternative measure of effect size, calculated by:

σm^2^ / (σm^2^ + σ^2^)

to estimate the sample size [21–23]. Generally, the effect size can be defined as follows: η^2^ = 0.0099 ≈ 0.01 is a small effect; η^2^ = 0.0588 ≈ 0.06 is a medium effect and η^2^ = 0.1379 ≈ 0.14 is a large effect [21–23]. In the study, we would like to determine the sample size required to detect a medium effect when the power is 0.90. Therefore, a total sample of 204 subjects (68 in each group) would be required. Assuming an overall 15% drop-out rate, 80 subjects should be recruited in each group. Finally, the total sample size is determined to be 240 patients.

### Data management

All CRFs in triplicate will be reviewed by the investigators and CRA. The completed CRFs will be securely stored in a locked location and finally sent to two independent data administrators, who will responsible for data entry, inspection and management in a specified statistics center. Once the trial is completed, the Principal Investigator, Sponsor, data administrators and statisticians will perform a blind review to confirm the dataset. The final database will be locked and analyzed in line with the statistical analysis plan.

### Statistical analysis

The independent statistician will conduct the data analysis using SAS 9.4 software (SAS, Cary, NC, USA), in accordance with the statistical analysis plan for this trial. The primary analysis set for efficacy is the full analysis set (FAS) with an intention-to-treat (ITT) principle, in which all patients treated with at least one dose of study drug and who have a clinical observation record should be involved. According to an ITT principle, the last-observation-carried-forward (LOCF) imputation method will be used for missing data. Patients who fulfil the protocol without any major deviations will be included in the per-protocol set (PPS). And all participants who receive at least one dose of the study drug and for whom safety data are available will be included the safety set (SS). Baseline characteristics of all subjects will be analyzed using descriptive statistics for continuous variables and categorical variables. Primary efficacy will be analyzed by comparing outcomes among three groups. Outcomes among three groups will be compared using analysis of variance (ANOVA) and the Bonferroni method. Covariates designated as potential confounders will include age, gender, body mass index (BMI) and duration of symptomatic illness prior to enrollment. Time to fever relief, time to fever clearance and duration of all symptoms will be estimated by the Kaplan-Meier technique and compared by the stratified log-rank test. The improvement of TUM symptoms will be estimated by descriptive analysis for number and proportion of patients achieving clinical recovery. Prespecified subgroup analysis and sensitivity analysis involving covariance analysis will be used to evaluate the primary outcomes according to age and gender. In the multicenter trial, the Cochran-Mantel-Haenszel (CMH) test will be performed to analyze stratified variables. A two-sided *P* value <0.05 is considered as statistically significant.

## Discussion

To date, this study is the first randomized, double-blind, placebo-controlled trial to evaluate the efficacy and safety of Uighur medicine in treating the common cold. In Central Asia, TUM is one of the most common traditional medicines, which has benefited many people in China and the other countries. So far, most of TUM researches have focused on therapy of skin disease, urogenital disease, rheumatism, digestive system and respiratory system disease [24]. To our knowledge, there have been few reports of standardized clinical trials designed for use in TUM.

Binafuxi formula has been used in Xinjiang Uygur Autonomous Region of China for over 20 years and based on local clinical experience it has been proven effective in treating CCHS. Derived from five different regions in China, the results of our study will provide stronger evidence for wider use of Binafuxi granules. We designed this study to practice the methodology of syndrome differentiation for clinical research in TUM. According to the grading standard for TCM, we will first perform the TUM symptom graded score system in this trial, and then verify if it is feasible. This will provide a scientific quantitative standard to estimate the efficacy of TUM in treating the CCHS. In the light of TUM theory on the CCHS and the characteristics of Binafuxi, the time to fever relief and the duration of fever will be primarily observed in this study [17]. The efficacy both of Western medicine and TUM will be evaluated, so as to provide more powerful evidence for TUM therapy for the common cold.

Nevertheless, there are two potential limitations in this study. During the screening period, we will not confirm viral titers to exclude potential patients with influenza since this would largely delay patient enrollment for the study and potentially miss the best time to intervene. However, all the experienced physicians involved for this study will carefully evaluate patients to avoid mistakenly recruiting unsuitable participants. In accordance with the exclusion criteria, we will strictly exclude any participants with body temperature > 39°C and severe respiratory symptoms. Patients with chills and/or other influenza-like symptoms will be excluded or advised to be tested for the influenza virus. Furthermore, in this study, we will not explore the mechanisms underlying the CCHS and potential pathways through which Binafuxi may work in treating the common cold, which may need further research.

In conclusion, this protocol is rigorously designed for exploring the efficacy and safety of Binafuxi granules in treating the common cold with heat syndrome. As far as we know, this study will be the first multicenter, double-blind, placebo-controlled and randomized clinical trial of a Uighur medicine in treating the common cold. In addition, it will provide robust evidence for assessment of the efficacy and safety of Binafuxi granules in treating the CCHS, and may help in selecting the optimal therapeutic dose for the next phase III clinical trial.

## Trial status

The study is currently in the process of recruiting participants. Recruitment of participants commenced on 5 February 2018 and will be completed in early May 2019. The protocol version number is Z-BNFX-GR-II-2017-YDL-01, dated 22 May 2017.

## Additional file

Additional file 1: SPIRIT 2013 checklist: recommended items to address in a clinical trial protocol and related documents. (DOC 134 kb)

## Abbreviations

AEs: Adverse events
ALP: Alkaline phosphatase
ALT: Alanine transferase
ANOVA: Analysis of variance
AST: Aspartate transferase
BMI: Body mass index
BUN: Blood urea nitrogen
CAM: Complementary and alternative medicine
CCHS: Common cold with heat syndrome
CRF: Case report form
ECG: Electrocardiogram
FAS: Full analysis set
GCP: Good clinical practice
GGT: Gamma-glutamyl transpeptidase
ITT: Intention to treat
LOCF: last observation carried forward
PPS: Per-protocol set
PSSR: Percentage of symptom score reduction
SFDA: State Food and Drug Administration
SOP: Standard operation procedure
SS: Safety set
TBil: Total bilirubin
TCM: Traditional Chinese medicine
TUM: Traditional Uighur medicine
USA: United States of America
